# Diffuse Cerebral Edema and Impending Herniation Complicating Hepatic Encephalopathy in Hereditary Hemorrhagic Telangiectasia

**DOI:** 10.1155/2022/2612544

**Published:** 2022-02-18

**Authors:** Charisma Mylavarapu, Alexander J. Lu, Ethan A. Burns, Justin Samorajski, Deepa Gotur, Kelty Baker

**Affiliations:** ^1^Houston Methodist Department of Medicine, Houston Methodist Hospital, 6550 Fannin St, Suite 1001, Houston, TX 77030, USA; ^2^Houston Methodist Cancer Center, Houston Methodist Hospital, 6445 Main St, Outpatient Center, Floor 24, Houston, TX 77030, USA; ^3^Houston Methodist Department of Radiology, Houston Methodist Hospital, 6565 Fannin St, Houston, TX 77030, USA; ^4^Houston Methodist Department of Pulmonary & Critical Care Medicine, Houston Methodist Hospital, 6550 Fannin St, Suite 1001, Houston, TX 77030, USA; ^5^Houston Methodist Hospital, 6560 Fannin St, Suite 1260, Houston, TX 77030, USA

## Abstract

Hereditary hemorrhagic telangiectasia (HHT) is an autosomal dominant vascular disease characterized by the formation of cutaneous and visceral telangiectasias and arteriovenous malformations (AVM). Multiple organs may be affected, including the nasal mucosa, skin, lungs, gastrointestinal tract, and brain. The following case highlights a unique manifestation of HHT in a patient with a gastrointestinal hemorrhage and epistaxis, resulting in hyperammonemia and diffuse cerebral edema and herniation. Clinicians should be aware of this potential complication in such patients and initiate ammonia-reducing agents early to avoid this devastating consequence.

## 1. Introduction

Hereditary hemorrhagic telangiectasia (HHT), eponymously known as Osler-Weber-Rendu syndrome, is an autosomal dominant disorder characterized by widespread angiogenesis. HHT has a wide geographic and ethnic distribution. In North America and Europe, the prevalence is estimated to be 1:10,000 [1, 2]. In geographically isolated populations, the prevalence of HHT is higher, such as an estimated 1:5000 in Japan [3] and 1:1330 in the Afro-Caribbean islands of Curaçao and Bonaire [4]. However, the true prevalence is likely underestimated, given its broad array of phenotypic presentations [5, 6].

HHT is a clinical diagnosis, which has been codified by the Curaçao Criteria (Table 1) [7]. The presence of recurrent epistaxis is the most sensitive clinical manifestation of HHT, with average onset of epistaxis at 12 years and nearly 100% of patients affected by age 40. However, visceral lesions, which are the greatest contributor to HHT mortality, are often undetected and asymptomatic. The mortality rate increases with age, with the cumulative death rate at age 50 estimated to be 14% in one study [8]. Thus, early HHT detection, screening, and intervention are essential for improving patient outcomes [9]. HHT can be diagnosed by looking for specific genotypic mutations. Over 97% of patients with HHT possess mutations in *ENG*, *ACVRL1*, or *SMAD4*, which modulate TGF-*β* signaling [9, 10]. However, diagnosis is not contingent on the presence of these mutations.

HHT's most life-threatening manifestations arise in the form of visceral arteriovenous malformations (AVMs), which may result in high-output heart failure, portal hypertension, and hepatic encephalopathy [11]. Gastrointestinal bleeding and hepatic dysfunction are relatively common, and cohort imaging studies have shown that over two-thirds of patients with HHT possess hepatic AVMs [12]. Hepatic AVMs in HHT rarely lead to hyperammonemia and resultant hepatic encephalopathy [13–19]. This case illustrates the presentation of the first reported occurrence of subsequent transtentorial herniation as well as management strategies.

## 2. Case

A 64-year-old female with HHT presented to the hospital with a 24-hour history of nonbloody diarrhea and new onset hematemesis. Her HHT was diagnosed 40 years prior and had been complicated by refractory epistaxis, recurrent gastric bleeding from gastric ulcers and AVMs, pulmonary arterial hypertension, and hepatic AVMs resulting in hyperammonemia and hepatic encephalopathy. She had been treated with steroids, danazol, doxycycline, aminocaproic acid, and bevacizumab and had undergone septodermoplasty and posterior nosebleed cauterizations. Most recently, she was receiving thalidomide 50 mg nightly with clinical improvement. She was also prescribed lactulose for her history of hepatic encephalopathy and iron supplementation. She had never smoked and did not drink alcohol.

**Figure 1:**
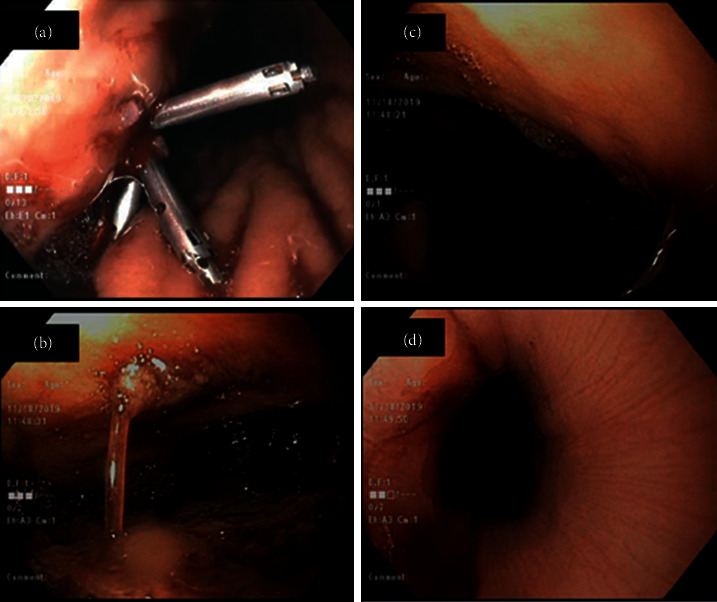
Endoscopy images showing gastrointestinal bleeding. (a) Bleeding gastric ulcer. (b, c) Large blood clots within the gastric body. (d) Blood in the lower third of the esophagus.

**Figure 2:**
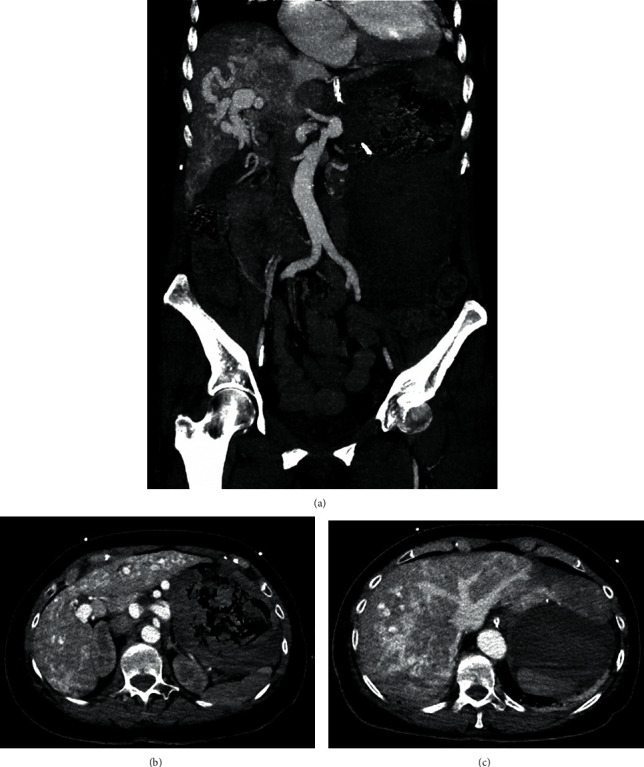
Coronal and axial late arterial phase abdominal CT imaging demonstrating hepatic arteriovenous shunting. (a) Coronal reconstruction showing hepatic arterial enhancement, the portal venous system is not opacified with contrast. There is early contrast filling of the hepatic veins and the inferior vena cava consistent with the hepatic arteriovenous shunting. (b) Axial slice through the level of the portal vein shows no contrast opacification of the portal system. (c) Axial slice through the level of the hepatic vein shows contrast enhancement. Incidentally noted is a markedly dilated stomach filled with blood products from gastrointestinal angiodysplasia.

On arrival to the emergency department, she had large volume hematemesis. She was hypotensive and tachycardic. On examination, she was lethargic but arousable. Her skin and conjunctiva were pale, and she had abdominal distension with generalized tenderness to palpation, along with melena and hematochezia on rectal exam. Her labs on admission were notable for a hemoglobin of 5.0 g/dL, platelet count of 103 k/*µ*L, prothrombin time of 15.4 seconds, fibrinogen of 149 mg/dL, and blood urea nitrogen (BUN) of 38 mg/dL. She had an ammonia level of 120 mmol/L. A nasogastric tube (NGT) was placed, and the patient was subsequently intubated for airway protection and transferred to the intensive care unit where she received numerous transfusions of packed red blood cells (pRBC), platelets, fresh frozen plasma (FFP), and cryoprecipitate. In addition, infusions of pantoprazole, octreotide, norepinephrine, and normal saline were given. Emergent esophagogastroduodenoscopy (EGD) performed at bedside showed a large volume of blood at the lower third of the esophagus and stomach, along with a few nonbleeding cratered gastric ulcers (Figure 1). Computed tomography angiography (CTA) was performed, demonstrating extensive arteriovenous (AV) shunting from underlying vascular malformations in the liver, though there was no localizable source of bleeding (Figure 2).

**Figure 3:**
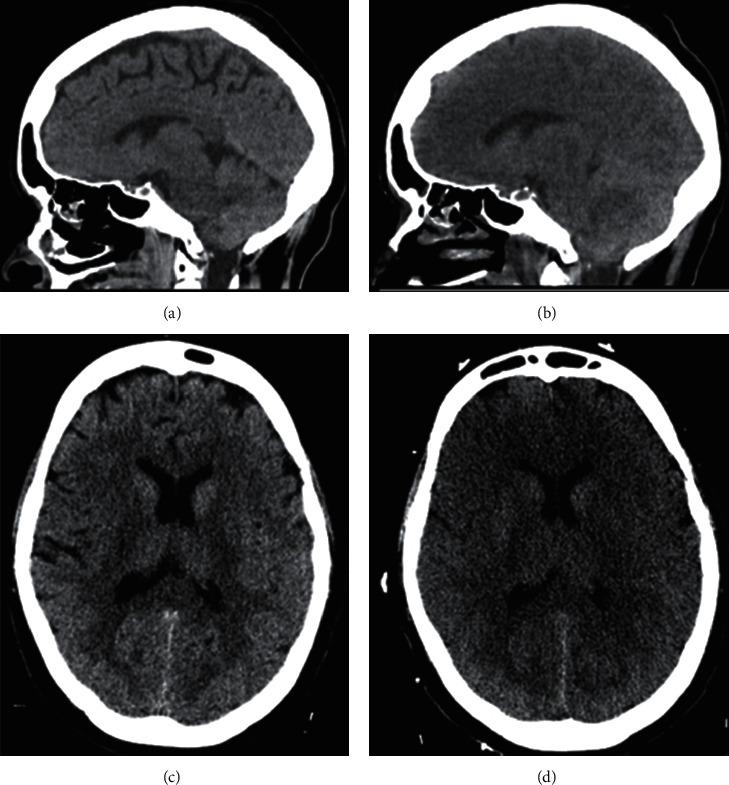
Sagittal and axial head CT performed before and 12 hours after the patient developed severe hyperammonemia. (a, b) Normal sagittal and axial brain CT. (c, d). Repeat head CT after clinical deterioration revealed diffuse hypodense cytotoxic edema with compression of the lateral ventricles and sulcal effacement. Sagittal view demonstrates interval compression of the subarachnoid cisterns and cerebellar tonsil herniation.

Within 24 hours of her arrival to the ICU, she developed hypertensive emergency, bradycardia, and tachypnea. On assessment, she had dysconjugate gaze, was minimally responsive to verbal or tactile stimuli, and had clonus with a positive Babinski sign bilaterally. Computed tomography (CT) head without contrast was performed but did not reveal acute intracranial abnormalities (Figures 3(a) and 3(b)).

**Figure 4:**
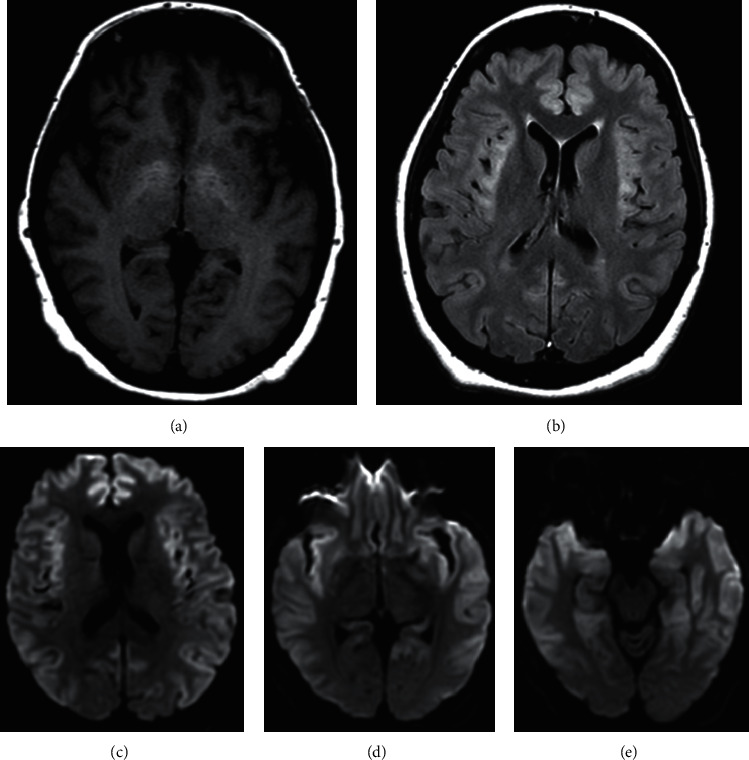
Slices of axial brain MRI performed 15 hours after CT head. (a) T1 sequence showing bilateral hyperintense basal ganglia characteristic of chronic hepatic encephalopathy. (b) T2 FLARE sequence showing bilaterally symmetric intensities of the frontal and insular cortex. (c–e) DWI sequence showing prominent gray matter diffusion restriction of the bilateral frontal, temporal, parietal, and insular cortices.

Ativan, propofol, and levetiracetam were administered for suspected subclinical seizures, and continuous electroencephalogram (cEEG) monitoring was started. The cEEG did not demonstrate evidence of active seizures but showed low-voltage activity with a severely depressed background consistent with a diffuse disturbance in brain function. The following day magnetic resonance imaging (MRI) was performed and suggested diffuse hypoxic injury (Figure 4).

The patient's ammonia level was measured and increased from 120 *µ*mol/L on admission to 259 *µ*mol/L, despite the administration of oral rifaximin and lactulose enemas. Repeat CT head without contrast performed twelve hours later showed diffuse cerebral edema (Figure 3). Although 3% hypertonic saline was started, serial CT scans demonstrated progressive worsening of the cerebral edema with impending transtentorial herniation (Figures 4(c) and 4(d)).

**Figure 5:**
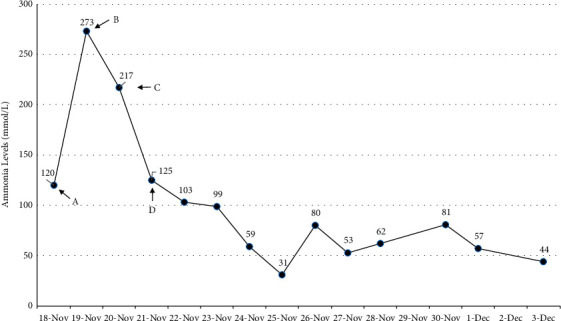
Trend of ammonia level over the course of this patient's hospital admission. (A) Ammonia level on admission. (B) Initiation of lactulose enemas and CRRT. (C) L-Arginine and sodium benzoate/sodium phenylacetate started. (D) CRRT stopped due to recurrent clotting within the machine.

Because her ammonia levels did not improve with pharmacologic management, she was started on renal replacement therapy (RRT) for the sole purpose of ammonia clearance, though this proved to have limited success. Eventually, a combination therapy of sodium benzoate, sodium phenylacetate, and L-arginine dropped her serum ammonia levels to a nadir of 31 *µ*mol/L (Figure 5). Ornithine and phenylalanine amino acid levels were within reference ranges, ruling out an inborn disorder of the urea cycle.

Continued bleeding precipitated disseminated intravascular coagulation. She required numerous blood products, and her bleeding slowly improved over the course of one week. While her ammonia normalized and serial imaging showed improvement in the cerebral edema, she remained in a persistent vegetative state and was ultimately transitioned to hospice.

## 3. Discussion

This case highlights the significant neurologic sequelae of uncontrolled gastric hemorrhage in an HHT patient with hepatic AVMs. The majority of HHT cases with hepatic involvement are asymptomatic, although some individuals can develop high-output cardiac failure, biliary ischemia, or portal hypertension [20]. There are reports published in the literature of patients with HHT and hepatic AVMs who develop portosystemic shunting, hyperammonemia, and subsequent encephalopathy. However, the patients in these cases responded to treatment with lactulose, without reaching the level of acute decompensation and cerebral herniation seen in this case [18, 21]. To our knowledge, this is the first reported case of hyperammonemia-induced transtentorial herniation occurring in HHT.

When blood is digested following gastrointestinal hemorrhage, globin breakdown results in a rise in circulating amino acids and ammonia which are subsequently converted to urea by hepatocytes in the liver via the urea cycle [22]. However, for patients with impaired hepatic function, the liver is unable to efficiently convert ammonia to urea, which results in a toxic accumulation of ammonia. Ammonia is able to cross the blood-brain barrier where it is converted to glutamine by glutamine synthetase within cerebral astrocytes. Glutamine accumulation within astrocytes leads to increased osmotic pressure and cerebral edema, putting patients at risk for elevated intracranial pressure and cerebral herniation [23].

Portosystemic encephalopathy due to hyperammonemia is rarely reported in HHT and has been attributed to the presence of hepatic AVMs. There are three categories of hepatic shunting that occur in patients with HHT, including those between the hepatic artery and hepatic vein; hepatic artery and portal vein; and the portal vein and hepatic vein [21]. Shunting between the portal vein and hepatic vein is the mechanism by which hepatic encephalopathy is thought to occur [16]. In patients with refractory GI bleeding and hepatic AVMs or cirrhosis, determining the optimum therapy to reduce a toxic accumulation of ammonia is paramount in reducing patient morbidity and mortality. Though there are no guidelines for RRT use in hyperammonemia, it appears to be a plausible treatment option [24]. However, as this case demonstrated, it may not always be efficacious. Another option is to facilitate removal of ammonia via the urea cycle. L-Arginine acts to increase the activity of *N*-acetylglutamate synthetase and carbamoyl phosphate synthetase-1, key enzymes within the urea cycle, by increasing the availability of L-citrulline and lessening hyperammonemia [25].

Sodium benzoate/sodium phenylacetate is a combination medication used in hyperammonemia. Its mechanism involves the conjugation of benzoate with glycine and phenylacetate with glutamine to yield hippuric acid and phenylacetylglutamine, respectively. Because glycine and glutamine contain nitrogen from ammonia, by combining these substances with benzoate and phenylacetate, their end-products can be renally excreted [26, 27]. These treatments substantially reduce ammonia levels.

## 4. Conclusion

This case uniquely demonstrates a neurologic catastrophe that may occur in patients with HHT who have hepatic AVMs and GI bleeding but also suggests potential treatment strategies that should be initiated early. Patients with hepatic AVMs may have a predisposition to encephalopathy after experiencing bleeding events, including GI bleeds and epistaxis. As this case illustrates, cerebral herniation can occur regardless of expedient management, but early treatment with amino acid therapy, RRT, rifaximin, and rectal lactulose should be considered.

## Figures and Tables

**Table 1:** Curaçao Criteria for clinical diagnosis of HHT.

Criteria^*∗*^	Description
Epistaxis	Spontaneous and recurrent
Telangiectasias	Multiple, at characteristic sites: lips, oral cavity, fingers, nose
Visceral lesions	Gastrointestinal telangiectasia, pulmonary, hepatic, cerebral, or spinal arteriovenous malformations
Family history	A first-degree relative with HHT according to these criteria

^*∗*^Presence of ≥3 criteria is “definitive for diagnosis” of HHT, 2 criteria is “possible or suspected” HHT, and 0 or 1 criteria is “unlikely” to be HHT. HHT: hereditary hemorrhagic telangiectasia.

## References

[1] Roman B. L., Marchuk D. A., Trerotola S. O., Pyeritz R. E. (2020). Hereditary hemorrhagic telangiectasia (Osler-Weber-Rendu syndrome). *Emery and Rimoin’s Principles and Practice of Medical Genetics and Genomics*.

[2] Shovlin C. L., Buscarini E., Kjeldsen A. D. (2018). European reference network for rare vascular diseases (VASCERN) outcome measures for hereditary haemorrhagic telangiectasia (HHT). *Orphanet Journal of Rare Diseases*.

[3] Dakeishi M., Shioya T., Wada Y. (2002). Genetic epidemiology of hereditary hemorrhagic telangiectasia in a local community in the northern part of Japan. *Human Mutation*.

[4] Westermann C. J. J., Rosina A. F., De Vries V., Coteau P. A. D. (2003). The prevalence and manifestations of hereditary hemorrhagic telangiectasia in the Afro-Caribbean population of The Netherlands Antilles: a family screening. *American Journal of Medical Genetics*.

[5] Shovlin C. L., Jackson J. E., Bamford K. B. (2008). Primary determinants of ischaemic stroke/brain abscess risks are independent of severity of pulmonary arteriovenous malformations in hereditary haemorrhagic telangiectasia. *Thorax*.

[6] Donaldson J. W., McKeever T. M., Hall I. P., Hubbard R. B., Fogarty A. W. (2014). The UK prevalence of hereditary haemorrhagic telangiectasia and its association with sex, socioeconomic status and region of residence: a population-based study. *Thorax*.

[7] Shovlin C. L., Guttmacher A. E., Buscarini E. (2000). Diagnostic criteria for hereditary hemorrhagic telangiectasia (rendu-osler-weber syndrome). *American Journal of Medical Genetics*.

[8] Sabba C., Pasculli G., Suppressa P. (2006). Life expectancy in patients with hereditary haemorrhagic telangiectasia. *QJM*.

[9] Faughnan M. E., Mager J. J., Hetts S. W. (2020). Second international guidelines for the diagnosis and management of hereditary hemorrhagic telangiectasia. *Annals of Internal Medicine*.

[10] Benzinou M., Clermont F. F., Letteboer T. G. W. (2012). Mouse and human strategies identify PTPN14 as a modifier of angiogenesis and hereditary haemorrhagic telangiectasia. *Nature Communications*.

[11] Garcia-Tsao G., Korzenik J. R., Young L. (2000). Liver disease in patients with hereditary hemorrhagic telangiectasia. *New England Journal of Medicine*.

[12] Buonamico P., Suppressa P., Lenato G. M. (2008). Liver involvement in a large cohort of patients with hereditary hemorrhagic telangiectasia: echo-color-doppler vs multislice computed tomography study. *Journal of Hepatology*.

[13] Guttmacher A. E., Marchuk D. A., White R. I. (1995). Hereditary hemorrhagic telangiectasia. *New England Journal of Medicine*.

[14] Govani F. S., Shovlin C. L. (2009). Hereditary haemorrhagic telangiectasia: a clinical and scientific review. *European Journal of Human Genetics*.

[15] Reagan T. J., Bloom W. H. (1971). The brain in hereditary hemorrhagic telangiectasia. *Stroke*.

[16] Garcia-Tsao G. (2007). Liver involvement in hereditary hemorrhagic telangiectasia (HHT). *Journal of Hepatology*.

[17] Ono K., Obara T., Takeshita M. (2017). A case of hereditary hemorrhagic telangiectasia with hepatic encephalopathy due to portal hepatic venous shunt. *Nippon Ronen Igakkai Zasshi. Japanese Journal of Geriatrics*.

[18] Shah R. N., Makar M., Akhtar N., Forster E. (2019). Lactulose to the rescue: a case of toxic hepatic encephalopathy caused by portosystemic shunting and epistaxis in a patient with hereditary hemorrhagic telangiectasia. *Case Reports in Hepatology*.

[19] Joughin D., McCanny P. (2020). Hereditary haemorrhagic telangiectasia: a rare cause of hepatic encephalopathy. *Journal of Anesthesia and Intensive Care Medicine*.

[20] Khalid S., Garcia-Tsao G. (2008). Hepatic vascular malformations in hereditary hemorrhagic telangiectasia. *Seminars in Liver Disease*.

[21] Buscarini E., Leandro G., Conte D. (2011). Natural history and outcome of hepatic vascular malformations in a large cohort of patients with hereditary hemorrhagic teleangiectasia. *Digestive Diseases and Sciences*.

[22] Damink S. W. M. O., Dejong C. H. C., Jalan R. (2009). Review article: hyperammonaemic and catabolic consequences of upper gastrointestinal bleeding in cirrhosis. *Alimentary Pharmacology & Therapeutics*.

[23] Tranah T. H., Paolino A., Shawcross D. L. (2015). Pathophysiological mechanisms of hepatic encephalopathy. *Clinical Liver Disease*.

[24] Gupta S., Fenves A. Z., Hootkins R. (2016). The role of RRT in hyperammonemic patients. *Clinical Journal of the American Society of Nephrology*.

[25] Liu J., Lkhagva E., Chung H.-J., Kim H.-J., Hong S.-T. (2018). The pharmabiotic approach to treat hyperammonemia. *Nutrients*.

[26] McGuire B., Al Sibae M. (2009). Current trends in the treatment of hepatic encephalopathy. *Therapeutics and Clinical Risk Management*.

[27] Mylavarapu C., Lu A. J., Burns E. A., Gotur D., Baker K. (2021). Cerebral herniation arising from hereditary hemorrhagic telangiectasia. https://shmabstracts.org/abstract/cerebral-herniation-arising-from-hereditary-hemorrhagic-telangiectasia/.

